# The Principles and Practice of Obstetric Medicine, in a Series of Systematic Dissertations on Midwifery, and on the Diseases of Women and Children

**Published:** 1833-10

**Authors:** 


*The Principles and Practice of Obstetric Medicine, in a Series of Systematic Dissertations on Midwifery, and on the Diseases of Women and Children. Illustrated by numerous Plates.* By David D. Davis, m.d., m.r.s.l., Professor of Midwifery in the University of London. Parts I. to XXII. London, 1833. 4to.

Although our predecessors have not neglected to notice Dr. Davis's work as its numbers issued from the press, yet, as we are now entering upon a fresh stage of existence, and as the science of obstetrics will, from a conviction of its paramount importance, occupy a considerable share of our attention, we shall probably gratify our new readers by noticing in the first number the work whose title stands at the head of this article.

To any one exercising the common powers of reflection, considerable astonishment must have been felt at the apathy and neglect with which, until of late years, the subject has been treated. We dare venture to assert, that, previous to the enforcement of attendance upon midwifery lectures by the College of Surgeons and the Apothecaries' Company, not one pupil in twenty paid any attention to this necessary department of his studies; and even now the regulations are very insufficient to secure that degree of information which ought to be possessed by every man before taking upon himself the responsibility attached to the practitioner of midwifery: for what, after all, do their rules amount to? Do they require the candidates to undergo a searching examination as the test of his qualification? Do they require that he shall not only have studied the theory, but that he should have had opportunities of putting his principles into practice in the lying-in room? Not they; nor, until very recently, was even an attendance upon the lectures required. It is true, a certificate of such attendance is now demanded; and what is proved by it? simply, that the pupil has paid a sum of money to an obstetric teacher for the purpose of "taking out his ticket." Knowing that he will not be examined upon any subject treated of by the lecturer, he, perhaps naturally, directs his attention to matters of minor importance, but which nevertheless "tell" at the college and hall. Notwithstanding, however, the want of encouragement which has attended the practitioners of midwifery, men have been found among them of no ordinary stamp; and we need only mention a few names, such as William Hunter, Smellie, Denman, Burns, Gooch, Haighton, Blundell, and Clarke, to prove the truth of this assertion. But why this neglect of what must be considered a very important part of professional knowledge? Why have not our medical colleges and corporations bestirred themselves in this matter? To those acquainted with the history of all chartered monopolies the secret is soon revealed. One common spirit appears to have actuated them from their earliest formation down to the present day, viz. the exclusive benefit of the privileged few, to the manifest disadvantage of the community at large. The regular physician, considering it as belonging to the department of surgery, refuses to practise it; the *pure* surgeon, knowing that all places of honour and of profit are closed to him if he practises midwifery, also disavows it, although it is a notorious fact that these very men will attend in consultation upon cases involving in their consideration a thorough acquaintance with the principles of obstetric science. It is greatly to be hoped, however, that in the inquiry concerning the state of medical education which will shortly take place, the evils attending the present system will be brought to light, and a more complete, just, and perfect one adopted. Now, however, to the subject more immediately before us.

Dr. Davis's work is published in monthly parts, twenty-two of which have already appeared. We are told that it will be completed in between thirty and forty numbers: judging from what has been printed, we should be inclined to suspect it will reach double that number, which will render it exceedingly expensive, when compared with our two standard works, Denman and Burns, neither of which, we prophesy, will be superseded by it; for, whilst the matter is by no means superior, the style is very decidedly inferior to either of them; and the expressions made use of by his reviewer in the "London Medical and Physical Journal" so exactly meet our views, that we assert with him that it is "a diffuse and inappropriate style of writing, which savours more of the efforts of a novel-writer to amuse his readers, than of a learned physician to instruct them." Some of the cases in illustration of particular subjects are related without much respect to delicacy; a most egregious failing in an obstetrical writer or teacher, whose grand aim should be to impress upon the minds of his readers or hearers, the necessity of avoiding every thing that can be construed into a want of the utmost delicacy and propriety of feeling.

Notwithstanding these deficiencies, Dr. Davis's work is valuable, not only as being the result of an extensive practice, but from the great number of references which are so frequently made to authors who have preceded him; so that the reader knows where to refer to without trouble, should he desire to be acquainted with the opinions of others upon any important disease. Dr. D. deserves great credit for his industry in this respect.

The arrangement of the subject is also good. Professor Burns has been followed in this respect, the anatomy of the parts concerned in the parturient act being first considered, and then the diseases to which these parts are subject; the osseous structure of the pelvis forming, as usual, the groundwork upon which alone can any accurate knowledge of the different varieties of labour be built. This part is especially deserving the student's attention. It would be quite as reasonable for a person to try to understand a language without knowing the alphabet, as to attempt the practice of midwifery without a thorough acquaintance with the shape, dimensions, and axis of the different parts of the pelvic cavity.

The chapter on "Diseased Conformations of the Pelvis" is well worthy the reader's attention.

Our author then proceeds to the external organs of generation, describing their "structures and diseases." We perfectly agree with the following observation respecting the *morbus pediculosus* of the mons Veneris: "There is no truth in the supposition, (a notion, however, which very generally prevails,) that the morbus pediculosus can only be acquired by sexual intercourse, or personal approximation in any other way to another individual previously affected with it;" facts proving the contrary having occurred in our own practice.

The treatment recommended in abscess of the labium is judicious; the recommendation of some authors not to interfere, but to allow it to burst of itself, being to our view very objectionable. There appears to be no good reason why the common treatment of abscess should in these instances be deviated from. We have been in the constant habit of making an early puncture, and are fully convinced that much time and uneasiness are saved to the patient by so doing.

Some interesting cases of enlargement of the labium from extravasated blood are related. The reader will do well to consult Burns' Midwifery upon this head; in a note appended to which he will find a number of references illustrative of this subject. It is highly necessary for the practitioner to be able to distinguish the causes which are giving rise to enlargement of the labium, which he will find to be numerous. Thus, in addition to that just mentioned, it may arise from common inflammation, from effusion into the cellular structure, either as a symptom of general anasarca, or it may be a local affection arising from the pressure of the gravid uterus interrupting the returning circulation; and, even in this latter affection, the size of the tumor will sometimes be very large, and materially interfere with the female's convenience. Where this is the case, relief will be experienced by making a few punctures with a clean sewing needle, through which the fluid will escape, and the tumor be consequently diminished; and the patient, by a repetition of the operation, if necessary, be rendered comfortable until after delivery. Enlargement of the labium may also arise from aneurism, from hernia, and from various kinds of tumors, fatty, encysted, scirrhous, &c.

The nymphæ are liable to many of the diseases which affect the labium, but the reader will find nothing of a novel nature upon this head in the work before us.

For the relief of virulent gonorrhœa, Dr. Davis advises a lotion composed of half a grain, or a grain, of oxymuriate of mercury in a pint of distilled water; which application was some years ago recommended by Dr. George Fordyce. The use of a mercurial wash, however, in the earlier stages of the disease, is objectionable. The local means which, in our own practice, have been found the most serviceable, consist in the assiduous application of a wash composed of half an ounce of tincture of opium, one drachm of Goulard's extract, and half a pint of distilled water; which, if properly applied, exerts an almost magical influence in allaying the ardor urinæ, which is the most distressing symptom of the disease. We are also at variance with the author respecting the general treatment, being convinced, from no inconsiderable experience, that "ample general bleedings" are seldom, if ever, required, brisk purgatives being much more efficient. After the subsidence of the actively inflammatory symptoms, astringent injections will be found more advantageous than the mercurial ointments recommended by Dr. Davis, especially when combined with the internal administration of one of the terebinthinate balsams.

The reader will find some very interesting observations upon the manner of introducing the catheter; and, in treating of the diseases of the urethra and bladder, a case is related in which a piece of wood had been forced up the urethra; and, although it "had been only fifteen days in the bladder, it exhibited on its surface a calcareous incrustation."

Pruritus of the external parts is a very troublesome and painful affection, which arises from various causes, and will therefore require a corresponding variety of treatment. Where its origin can be traced to herpetic eruptions, a solution of the nitrate of silver, dissolved in an ounce of distilled water, will seldom fail in procuring relief, and in many instances has succeeded in effecting a perfect cure, after other means has failed. This application is not noticed by Dr. D.

The experience of the author unfortunately coincides with our own respecting the uncertainty of affording relief in those fistulous openings which sometimes occur between the bladder and vagina. He observes:

"Various contrivances have been suggested to plug up the artificial passage between the bladder and vagina; such as globular bodies and pessaries of various forms and materials, sometimes hollowed out, and at other times solid: some smooth, others resisting, or elastic, yielding, mouldable, absorbent, &c.: but the author does not recollect a single example of the means in question having effected a permanent cure, where the intermediate aperture had been the result of sloughing.

"The practice of bringing the edges of such breaches of continuity together, and connecting them by means of ligatures, has in like manner very seldom been attended with success. The author does not wish to attach to either of these modes the discredit of having universally failed. But he is very much disposed to believe that in by far the greater number of cases in which they are represented to have been successfully employed, either the injuries for which they were used were trifling as to the magnitude of the original wound, or else the wound had been the effect of penetration by an edged or angular portion of fœtal bone, rather than of long-continued pressure and contusion; or, finally, the incontinence might be the result simply of reduced power, or possibly of reduced substance of the sphincter of the bladder, rather than that of a communication between the bladder and the vagina, in consequence of any variety of solution of continuity. A well-adapted globular body of proper size to admit a suitable part of its convex surface to be accurately adjusted to the boundaries of the aperture; capable also of some modification of its figure for the greater convenience of introduction and adjustment; readily chargeable with air for the purpose of distention, but nevertheless admitting of being made perfectly air-tight; so smooth on every part of its surface as to be easily tolerated when applied to the parts intended even in their most tender state: such an instrument might in many, perhaps in the majority of cases of intercommunications between the bladder and the vagina, be safely recommended, as a means of relief or mitigation of the distressing evils consequent upon the accession of so grievous a calamity. But no mechanical contrivance, nor any mode of management that has hitherto been proposed or adopted, can be relied upon as an absolute remedy." (P. 127.)

The various diseases of the vagina are clearly detailed, and the respective methods of treatment recommended upon sound and scientific principles.

The attention of the reader is earnestly directed to one very important part of this section, viz. on "hernial protrusions of intestines forming tumors within the vaginal passage." The necessity of discriminating between hernial and other tumors is great; not only the patient's comfort, but her life, might be sacrificed by an error in this respect. The importance of this branch of his subject has not been overlooked by Dr. D., who has consequently devoted a large space to the consideration of it.

Cystocele, or hernia of the bladder, is not frequently met with: it may take place either independently of, or connected with, pregnancy. If it occur at the time of labour, care must be taken thoroughly to evacuate its contents, and to endeavour to return it to its natural situation, by making steady pressure with two fingers, in the interval of the pains, so as to push it up beyond the head of the child, and keep it out of the way of pressure. The different kinds of cystic protrusion are described at length in the work before us.

The anatomy and function of the unimpregnated uterus form the next subject for consideration, and several curious cases of precocious menstruation are given on the authority of different writers. It is a well-known fact that menstrual evacuation generally makes its appearance when the female has arrived at the age of fifteen, or thereabouts, and that its cessation takes place at about forty-five; but Dr. Davis transcribes a case from the Transactions of the French Academy for 1768, which exhibits a remarkable deviation from the general rule. The subject of the communication was ninety-one years of age, and was then "subject to the ordinary evacuation of her sex;" and this discharge had continued regularly since the age of seventeen, excepting the periods of gestation and lactation, for she had borne and suckled twelve children. We quite concur in the opinion that "genuine menstruation has really never existed during gestation," although similar discharges of blood are by no means uncommon during that period, and are often the precursors of premature delivery. The following explanation is, to say the least, very ingenious.

"If the uterine branches of the internal iliacs be in a state of extraordinary fulness and development during gestation, is it not to be expected, nay, is not the fact obvious and undeniable, that its great sister branches, the vaginal arteries, should also sustain if not an equal, at least a very great enlargement of their capacity? It has been already seen how numerous are the inosculations of the several branches of the uterine epigastric arteries. Certain channels from a general reservoir being obstructed and securely made up, is it not perfectly intelligible, how other channels not similarly guarded may be made the subjects of new determinations and of unusual pressure of momentum, so as occasionally to be forced to yield to the extraordinary impetus made against them, and thus become the seats of transudations and floodings of similar character, and perhaps of not inferior importance to those of their contiguous and more considerable channels before the date of their supposed security? Such, in fact, is the explanation of the sanguineous discharges, at least when actually periodical, which take place during pregnancy. The natural outlet of the uterus is made up and fortified. No discharges can therefore take place from the arteries which administer to the operations carrying on within its cavity, without a violent removal of the flood-gate or plug, which nature has thrown across the narrow passage out of it." (P. 254.)

The supposed causes of menstruation are detailed at large. In a practical work, such unprofitable speculation should be avoided as much as possible; its only use (if use it may be called,) being that of increasing the size of the volume.

The disordered conditions of this function form very interesting and necessary objects of study. Every practitioner of midwifery knows the importance which is attached by females to the regular performance of the menstrual secretion, attributing in fact every ailment which they may at the time experience to the absence of it. This opinion has been productive of great mischief, inasmuch as it has led them to trust to empirical remedies, with the express intention of forcing, or "bringing down," as they term it, this discharge; whereas, in a very large majority of cases, if a proper investigation be had recourse to, it will be found that the absence of the catamenial flow is not the disease itself, but a mere symptom of it, and that consequently, in our curative attempts, the uterus must by no means be the only organ to which attention is to be directed.

In profuse chronic menorrhagia, the condition of the liver ought to be carefully inquired into; a free flow of blood through which is so necessary to the preservation of the healthy balance of the circulation. Congestion in this organ is the well-known cause of some of the worst cases of hemorrhoids, and we believe many long-continued discharges of blood from the womb may also be attributed to the same cause.

Dr. Davis's remarks on dysmenorrhœa and leucorrhœa deserve a very careful perusal. Many instances are related, showing the ill effects of suddenly suppressing leucorrhœal discharges which have existed for any length of time, and clearly proving the necessity of having recourse to internal in addition to topical measures of relief. The difficulty of distinguishing leucorrhœa from gonorrhœa is noticed, and we entirely agree with the author's conclusions; and, as the peace and happiness of families not unfrequently depend upon the opinion given by the medical man concerning the nature and cause of the discharge, we shall transcribe them for the benefit of our readers.

"It has been often a great desideratum in the profession to establish a satisfactory diagnosis between the matter of gonorrhœa, the known or suspected result of impure sexual intercourse, and the produce occasionally of some of the severer forms of leucorrhœa. It has been supposed that the accession of an irritating discharge from the vagina in a case never before the subject of any discharge at all, supervening upon a sexual congress, within the usual time and after the usual manner of a gonorrhœal affection, could be no other than the actual result of infection from that source. But it is a well-known fact, that the mechanical irritation attendant upon the first conjugal embrace is sometimes followed by a leucorrhœal secretion so abundant, so painful in its accompaniments, and in all respects so similar in its sensible properties to the matter of virulent gonorrhœa, as not to be distinguishable from it. The author has known many married ladies, the wives of perfectly healthy husbands, who were never subjects of leucorrhœa before marriage, and who have never been free from it subsequently. It has been a notion of some writers that gonorrhœa alone is accompanied by extreme phlogosis of the surfaces forming the vaginal orifice, and especially by the symptom called ardor urinæ. Both these statements are unfounded in fact. De Graaff, de Mulier. Organ. p. 140, supposes that we may distinguish fluor albus from venereal gonorrhœa by the respective localities of their sources; the former deriving its origin from the uterus, and the other being exclusively produced by a morbid secretion from the surfaces which form, or are immediately within the genital sulcus; as those of the external orifice of the vagina, of the external orifice of the urethra, of the clitoris, nymphæ, &c. In refutation of this opinion it need only be observed, that the surfaces just enumerated are liable to inflammatory affections, not to be distinguished in their appearance from similar conditions of parts produced by gonorrhœal infection, though certainly never exposed to the action of that virus, and often perfectly well known to be the results of other causes of irritation. Authors have sometimes attempted to discover the means of a diagnosis between the discharges incident, respectively, to gonorrhœal and leucorrhœal inflammations of the mucous membranes of the genital surfaces, from supposed differences in their colour, odour, density, and quantity. Baglivi, Prax. Medic. lib. ii. cap. 8, art. 3, propounds, as a principle of diagnosis in these cases, the assumed fact, that the leucorrhœal discharge ceases during menstruation, and *vice versa.* The experiments of Swiediaur and others, and the experience generally of the profession, and especially of accurate modern surgeons and pathologists, have abundantly proved the futility and unsatisfactoriness of all these distinctions." (P. 349.)

The propriety of "general bleeding" for the cure of leucorrhœa may very fairly be questioned; for, even in cases which are attended with an inflammatory state of parts, it will commonly be found that the profluvium itself is quite sufficient to relieve it; and, if not, the application of leeches is a preferable method.

After noticing the different hypotheses respecting the nature of hysteria, Dr. Davis very justly remarks:

"The most characteristic phenomenon incident to this remarkable disease is that of the convulsions by which its subjects are agitated. Were all its other properties to present themselves at the same time in the same individual, unaccompanied by this, they would scarcely be recognised as constituting a case of hysteria. If we examine closely the character of the greater number of premonitory, concomitant, and consecutive symptoms of an attack of hysteria, we shall not find it difficult to refer a very considerable proportion of its phenomena to the head as their principal source, the disturbances manifested by the thoracic and abdominal viscera being almost always consequences of the violent spasms which characterize the malady.

"If to that consideration we add that many subjects of hysteria, in its most violent forms, are often remarkable for possessing, during the intervals between its paroxysms, a good state of their chylopoietic functions, and that they exhibit the appearance of excellent general health, with much freshness of complexion; that the ordinary consequences of hysteria, when, after becoming a disease of many years' duration, such consequences follow, are most frequently lesions of the understanding, of the senses, and of the organs of voluntary motion; that, at the commencement of the malady, the organs of nutrition rarely exhibit any remarkable disorders of a permanent character; that hysteria has sometimes been seen complicated with epilepsy and catalepsy; that a great proportion of the causes of the disease are intense moral affections: if all these circumstances are really as they are stated, then is the hypothesis which refers the disease to derangements of the cerebral system as its principal and primary source, obviously entitled to much consideration. But, although hysteria might very truly have for its source certain morbid conditions of the brain and spinal marrow in the first instance, it is nevertheless a fact of so little importance, that in the sequel the viscera of the thorax and abdomen become frequently seats of lesions deserving of the most serious attention of the physician." (P. 398.)

And again,

"The hypothesis of its origin in some morbid but unknown condition of the cerebral system, may perhaps upon the whole deserve to be considered as the most probable. The reader, however, will be kind enough to observe, that all the speculations which have yet been offered on the subject have amounted to little more than to simple statements of opinions." (P. 400.)

The effect of a very powerful impression on the mind in preventing a fit of hysteria does not pass unnoticed, and the following curious circumstance is related from Boerhaave:

"In the poor's-house in the city of Haerlem, a young girl, greatly terrified, fell into a violently convulsive disease, which returned in successive paroxysms. One of the spectators and assistants, perhaps the most devoted to the performance of her friendly offices to the patient, was seized by the same disease. On the following day another was seized in the same way, and presently a third and a fourth, and at length nearly all the occupants of the ward, boys as well as girls. A most miserable state! observes the learned reporter of the scene. One was seized here, a boy; another was seized there, a young female. The one observed the fate of the other, and all, or nearly all, became prostrate together. The most skilful physicians had recourse to the exhibition of the most approved anti-epileptic medicines in vain. It was at length determined to seek the assistance of the celebrated Boerhaave, who, pitying the unhappy lot of the pauper establishment at Haerlem, was induced to go over to pay it a visit. On examining into the matter, he observed that, on the invasion of a paroxysm of the disease in the case of one of the patients, many others were immediately seized with similar convulsions. The best remedies having already been exhibited in vain by very intelligent practitioners, who had very duly recognised the ordinary influence which the imagination seemed to have exerted in propagating the malady from one person to another, that eminent physician nevertheless believed that, by restraining the operation of that influence, he might still be able to obtain a cure; and he did obtain a cure.

The magistrates of the city having been privately advised of his intention, and being all assembled, he ordered portable furnaces to be placed in different parts of the gallery, and strong fires to be kindled in them, and to be well fed and kept up by the most rapidly burning firewood. Into these furnaces he then ordered iron hooks of a particular shape to be thrust, in order to be made red hot. When all was ready, he spoke loudly to the following effect: 'Since all other means have fallen short of our object, I know of only one more remedy to propose, which however I expect will prove an infallible one, viz. that of applying one of these red-hot implements to the naked arm, and carried down to the bone of the first young man or woman who shall be seized with a paroxysm of this cruel disease.' As the whole of this address was enunciated with the utmost gravity, the inmates of the establishment were one and all much terrified, and stood aghast at the idea of a remedy so barbarous. The stronger impression, the fear of the pain of being burnt, prevailed over the influence of the extraordinary sympathy which had hitherto been competent to propagate the disease; and no person, either male or female, became on that occasion the subject of a paroxysm." (P. 421.)

Dr. Davis's treatment is upon the whole judicious, though we are no advocates for the abstraction of blood to the amount of from "twenty to five-and-thirty ounces" in any case; and, in by far the greater number of instances, venesection is not required at all, the tumult of the circulation being more readily subdued by what are usually termed antispasmodics. The state of the bowels should be carefully attended to, as hysterical paroxysms may be caused simply by an accumulation of fæces in the larger bowels.

The subject of nymphomania is discussed at very tedious length, and in a way not calculated to reflect much credit on the author. The chapter consists almost entirely of a tissue of indelicate (might we not say indecent) tales, which we should be very sorry to see transcribed on our pages, and therefore shall at once dismiss the topic.

We shall take leave of Dr. Davis for the present, intending in future to notice the different parts as they are published.

